# Karyotypic divergence reveals that diversity in the *Oecomys paricola* complex (Rodentia, Sigmodontinae) from eastern Amazonia is higher than previously thought

**DOI:** 10.1371/journal.pone.0241495

**Published:** 2020-10-29

**Authors:** Willam Oliveira da Silva, Celina Coelho Rosa, Julio Cesar Pieczarka, Malcolm Andrew Ferguson-Smith, Patricia Caroline Mary O’Brien, Ana Cristina Mendes-Oliveira, Rogério Vieira Rossi, Cleusa Yoshiko Nagamachi

**Affiliations:** 1 Laboratório de Citogenética, Centro de Estudos Avançados da Biodiversidade, Instituto de Ciências Biológicas, Universidade Federal do Pará (UFPA), Belém, Pará, Brazil; 2 Department of Veterinary Medicine, University of Cambridge, Cambridge Resource Centre for Comparative Genomics, Cambridge, United Kingdom; 3 Laboratório de Ecologia e Zoologia de Vertebrados, Instituto de Ciências Biológicas, Universidade Federal do Pará (UFPA), Belém, Pará, Brazil; 4 Departamento de Biologia e Zoologia, Instituto de Biociências, Universidade Federal do Mato Grosso (UFMT), Mato Grosso, Brazil; Tulane University Health Sciences Center, UNITED STATES

## Abstract

The genus *Oecomys* (Rodentia, Sigmodontinae) is distributed from southern Central America to southeastern Brazil in South America. It currently comprises 18 species, but multidisciplinary approaches such as karyotypic, morphological and molecular studies have shown that there is a greater diversity within some lineages than others. In particular, it has been proposed that *O*. *paricola* constitutes a species complex with three evolutionary units, which have been called the northern, eastern and western clades. Aiming to clarify the taxonomic status of *O*. *paricola* and determine the relevant chromosomal rearrangements, we investigated the karyotypes of samples from eastern Amazonia by chromosomal banding and FISH with *Hylaeamys megacephalus* (HME) whole-chromosome probes. We detected three cytotypes for *O*. *paricola*: A (OPA-A; 2n = 72, FN = 75), B (OPA-B; 2n = 70, FN = 75) and C (OPA-C; 2n = 70, FN = 72). Comparative chromosome painting showed that fusions/fissions, translocations and pericentric inversions or centromeric repositioning were responsible for the karyotypic divergence. We also detected exclusive chromosomal signatures that can be used as phylogenetic markers. Our analysis of karyotypic and distribution information indicates that OPA-A, OPA-B and OPA-C are three distinct species that belong to the eastern clade, with sympatry occurring between two of them, and that the “*paricola* group” is more diverse than was previously thought.

## Introduction

The arboreal genus *Oecomys* (Rodentia, Sigmodontinae) currently comprises 18 recognized species distributed from southern Central America to southeastern Brazil in South America, and is the most speciose genus of the Oryzomyini tribe. However, the actual number is uncertain, considering that morphological, phylogenetic (mtDNA and nuDNA), classic cytogenetics and chromosome painting analyses have shown that there is wide-ranging diversity within some lineages [1–6]. Although these multidisciplinary approaches have helped researchers to comprehend better the distribution range and taxonomy of *Oecomys*, the resolution of *O*. *bicolor*, *O*. *catherinae*, *O*. *cleberi*, *O*. *mamorae*, *O*. *paricola* and *O*. *roberti* remains controversial. These taxa have been proposed to constitute species complexes, but additional sampling with more accurate analysis are required to distinguish the actual evolutionary units [1,4].

**Fig 1:**
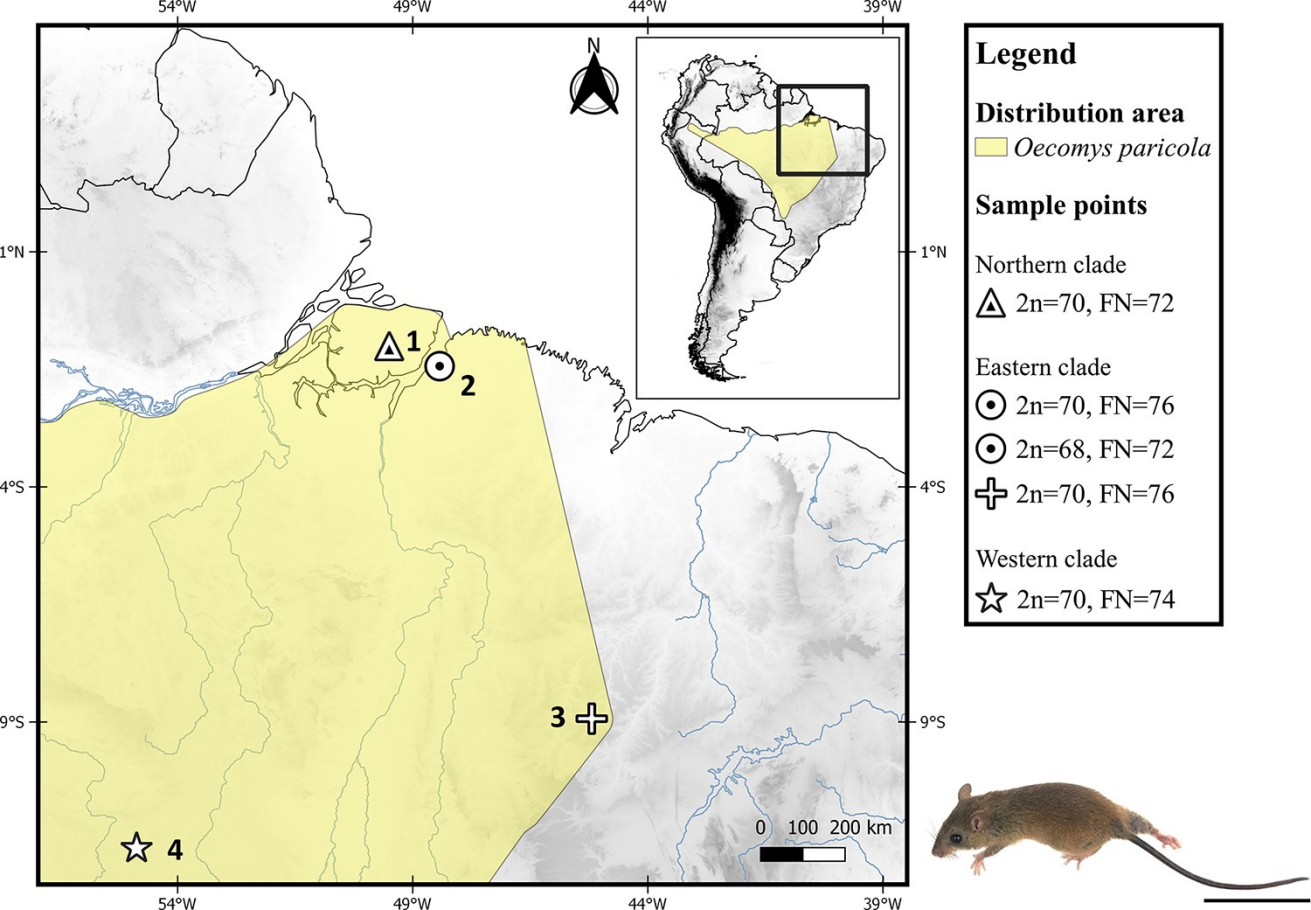
Map showing the distribution area and sampling points for *Oecomys paricola* with available cytogenetic data from the literature. 2n (diploid number), FN (autosomal fundamental number) and the clades identified for *O*. *paricola* (northern, eastern and western; sensu [4]) are also shown. The localities mentioned are: Tauarí farm, Chaves, Marajó Island—PA (locality 1); Utinga Reserve, Belém—PA (locality 2); Uruçui-Una—PI (locality 3); Cláudia—MT (locality 4). All localities are from Brazil, and from the states of Mato Grosso (MT), Pará (PA), and Piauí (PI). The map was made using QGIS v. 3.10.7. Geographic distribution of *Oecomys paricola* is based on sample points provided by Patton et al. [1] and Suárez-Villota et al. [4]. The database was obtained from DIVA-GIS [7]. The shapefiles data containing countries limits, hydrography and elevation were obtained in the link https://www.diva-gis.org/gdata. In this link we selected the shapefiles from every country that is showed in both Figs 1 and 5. An *O*. *paricola* specimen is shown below. Scale bar: 5 cm. Photo by WOS.

In particular, *O*. *paricola* exhibits a wide and uncertain distribution in central Brazil, from south of the Amazonas River to northeastern Peru [1] (Fig 1). This group was recovered as monophyletic by Suárez-Villota et al. [4], with high support values in maximum likelihood (ML) and Bayesian inference (BI) analyses performed with the concatenated genes, *Cytb*, *IRBP* and *iBF7*. The authors also recovered three distinct evolutionary lineages named the northern, eastern and western clades with high support within *O*. *paricola*, suggesting that the group represents a complex of different species. Beyond molecular identification, these evolutionary units can also be discriminated based on diploid number (2n), autosomal fundamental number (FN) and morphological traits [4]. The northern clade contains samples from the Marajó Island, north of Brazil in the Amazon biome, with 2n = 70, FN = 72 (locality no. 1 in the Fig 1); the eastern clade includes samples from Belém, north of Brazil in the Amazon biome, with 2n = 70, FN = 76 and 2n = 68, FN = 72 (locality no. 2 in the Fig 1), and specimens from the Cerrado biome in the states of Piauí, Maranhão and Tocantins, with 2n = 70, FN = 76 (locality no. 3 in the Fig 1); and the western clade includes samples from the state of Mato Grosso, with 2n = 70, FN = 74 (locality no. 4 in the Fig 1), south of the Amazon biome in central Brazil.

The level of information obtained from classic cytogenetics alone by using conventional staining, C-banding, and G-banding is lower than that achieved by FISH (fluorescence *in situ* hybridization) with whole-chromosome painting probes. The latter approach provides a better resolution of chromosomal evolution at many taxonomic levels, ranging from differences among populations of the same species and detection of complex rearrangements such as translocations and pericentric inversions [8–12], to the identification of conserved chromosomal segments among distinct mammalian groups that can be used as phylogenetic markers [10,13–19]. Furthermore, comparative chromosome painting analysis has been used as a valuable method to delineate species limits. In rodents, the information obtained by this approach has helped to elucidate the diversity within some groups and to establish geographic boundaries [12,20,21].

In the genus *Oecomys*, Malcher et al. [2] and Suárez-Villota et al. [4] used a multi-pronged approach to analyze samples of *O*. *catherinae*. By employing karyotypic, morphological and molecular methods, the groups reached similar conclusions, both proposing that *O*. *catherinae* populations from the Amazon (2n = 62, FN = 62) and Atlantic Forest biomes (2n = 60, FN = 62) are two distinct species. Although the two taxa from Amazonia and Atlantic Forest exhibited a low intraspecific molecular divergence of 1.6–3.1% [4] and 1.7% [2], they exhibited some distinct morphological characteristics. Suárez-Villota et al. [4] used C-banding analysis and proposed that the karyotypic divergence was due to one fission event, while Malcher et al. [2] used C-banding, G-banding and chromosome painting with HME (*Hylaeamys megacephalus*) whole-chromosome probes [22] and showed that the divergences were caused by one translocation and one fusion/fission event.

Taking into account the diversity observed in the “*paricola* group”, we set out to investigate the karyotypes of *O*. *paricola* from four localities of eastern Amazonia, aiming to improve their taxonomic delineation and determine the chromosomal rearrangements in this group. Toward this end, we performed a comparative analysis through classical cytogenetics and chromosome painting using HME whole-chromosome probes [22]. We also compared the taxa from the present study with other species from previously published studies using HME whole-chromosome probes [2,12,19,20,22–24].

Here, we reveal the chromosomal rearrangements that distinguish samples of *O*. *paricola* and describe exclusive chromosomal signatures for this taxon. We also propose that the samples in the present study correspond to three distinct species and discuss how chromosomal rearrangements may have played a part in the speciation process.

## Materials and methods

### Samples

The specimens were collected using live animal traps (Sherman) and pitfall traps [25], and the captures were authorized by the Brazilian Environment Department under license (IBAMA 02047.000384/2007-34). JCP has a permanent field permit (number 13248) from the Instituto Chico Mendes de Conservação da Biodiversidade. The specimens were deposited at the zoological collections of Museu Paraense Emílio Goeldii (MPEG) and the Museu de Zoologia da Universidade Federal do Pará (MUFPA). Both institutions are in Belém, Pará state, Brazil (Table 1). The Comitê de Ética em Pesquisa Animal da Universidade Federal do Pará approved this research (Permit 68/2015).

**Table 1:** Cytogenetic data available in the literature and obtained in the present study for *Oecomys paricola*. Species analyzed with *Hylaeamys megacephalus* probes in the present study are highlighted in bold in the leftmost column. The clades identified for *O*. *paricola* (northern, eastern and western; sensu [4]) are also shown within parentheses. Localities 1–4 refer to those mentioned in Fig 1; localities 2 and 5–7 are from the present study. Brazilian (BR) states are Mato Grosso (MT), Pará (PA) and Piauí (PI). The museum numbers of specimens analyzed in the present study are provided. Abbreviations: diploid number (2n); autosomal fundamental number (FN); Museu Paraense Emílio Goeldii (MPEG); Museu de Zoologia da Universidade Federal do Pará (MUFPA); male (♂); female (♀).

Species	Karyotype	Locality	Museum Number	Reference
*O*. *paricola* (Northern clade)	2n = 70/FN = 72	(1) BR, PA: Tauarí farm, Chaves, Marajó Island (00°39’S 50°11’W)		[26]
*O*. *paricola* (Eastern clade)	2n = 70/FN = 76	(2) BR, PA: Utinga Reserve, Belém (01°25’48.35”S 48°25’41.74”W)		[26]
*O*. *paricola* (Eastern clade)	2n = 70/FN = 76	(3) BR, PI: Uruçui-Una (8°55’38.54”S 45°11’35.07”W)		[4]
*O*. *paricola* (Western clade)	2n = 70/FN = 74	(4) BR, MT: Cláudia (11°41’4.49”S 54°52’21.22”W)		[4]
***O*. *paricola* cytotype A** (Eastern clade)	2n = 72/FN = 75*	(2) BR, PA: Utinga Reserve, Belém (01°25’48.35”S 48°25’41.74”W)	MPEG 39699♂, MPEG 39703♀	Present study
***O*. *paricola* cytotype B** (Eastern clade)**	2n = 70/FN = 75	(5) BR, PA: Expedito Ribeiro Community, Santa Bárbara (01°13’02.31”S 48°16’33.63”W)	MUFPA 377♂, MUFPA 372♀	Present study
		(6) BR, PA: Barcarena (01°31’13.12”S 48°41’25.09”W)	MUFPA 2100♂, MUFPA 2101♂, MUFPA 2102♀, MUFPA 2103♂, MUFPA 2104♂, MUFPA 2105♀, MUFPA 2106♀	
***O*. *paricola* cytotype C** (Eastern clade)**	2n = 70/FN = 72	(7) BR, PA: Tapirapé-Aquiri National Forest, Marabá (05°46’26”S 50°30’43”W)	MPEG 39892♂, MPEG 39894♂, MPEG 39906♂, MPEG 39907♂, MPEG 39908♀	Present study

*Previously assigned with 2n = 68/FN = 72 [26] but corrected to 2n = 72/FN = 75.

**The designation of the phylogenetic clade (sensu [4]) was based on distribution and karyotypic data from the present study.

### Cytogenetics

The cells used to prepare metaphase chromosomal preparations were obtained from bone marrow extraction performed according to Ford & Hamerton [27] and by cell culture of skin biopsy performed as described by Morielle-Versute [28]. The C-Banding [29] and G-Banding [30] techniques used slides bearing chromosomal preparations. C-banding was performed on G-banded metaphases to enable correct chromosomal assignment. FISH experiments followed Yang et al. [31] and were performed with 24 whole-chromosome probes from *Hylaeamys megacephalus* (HME) [22] made by degenerate oligonucleotide primed PCR (DOP-PCR) of flow-sorted chromosomes [31,32], of which three corresponded to two pairs of HME chromosomes each (HME (9,10), (13,22) and (16,17)). The labeling was made either with biotin-16-dUTP (Boehringer Mannheim), fluorescein isothiocyanate (FITC)-12-dUTP (Amersham) or Cy3-dUTP; the detection of the biotin probes was made with avidin-Cy3 or avidin-FITC.

The slides with chromosomal preparations were denatured for 2 minutes at 70% formamide, 2×SSC at 65°C for 1 minute; the HME probes were denatured for 15 minutes at 60°C before adding to the slides. After 72 hours hybridization at 37°C, the slides were washed (2x formamide 50%, 2x (2xSSC), 1x (4xSSC/Tween) at 40°C) [31,32]. The detection of the biotin probes was made with avidin-Cy3 (red) or avidin-FITC (green); for identification of the chromosomes pairs the counterstaining was made with DAPI (4',6-diamidino-2-phenylindole; blue).

### Image capture and analysis

Digital images of banded karyotypes were gathered using an Olympus BX41 microscope and a CCD 1300QDS digital camera, and analyzed using the GenASIs software v. 7.2.7.34276. FISH images were obtained using a Nikon H550S microscope and a DS-Qi1Mc digital camera, and analyzed using the Nis-Elements software. The karyotypes were organized according to Levan et al. [33], with modifications. The final images were edited using Adobe Photoshop CS6.

## Results

### Classic cytogenetics

**Fig 2:**
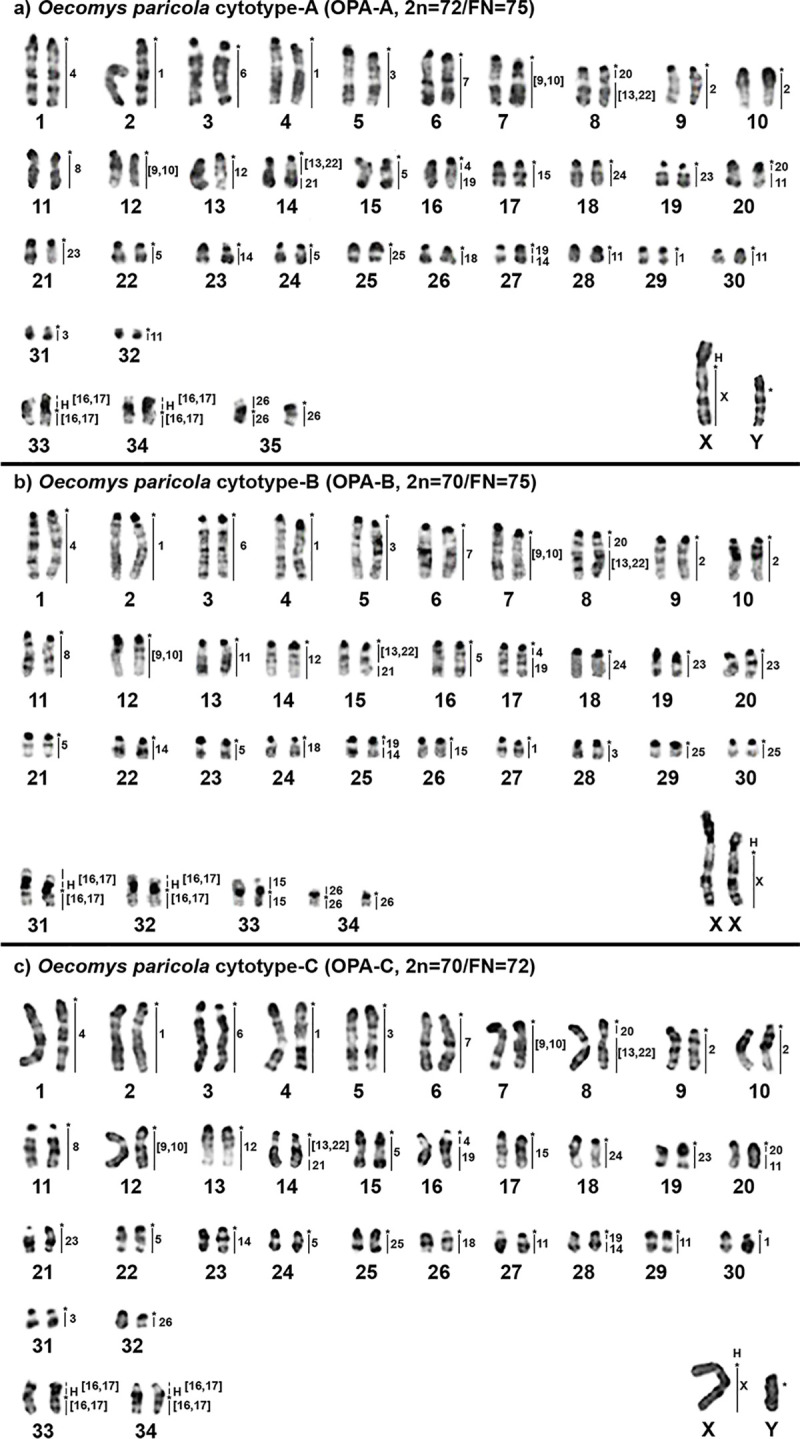
G-banded karyotypes with chromosome painting revealed by *Hylaeamys megacephalus* (HME) whole-chromosome probes [22] of (a) *O*. *paricola* cytotype A (OPA-A; 2n = 72/FN = 75), (b) *O*. *paricola* cytotype B (OPA-B; 2n = 70/FN = 75) and (c) *O*. *paricola* cytotype C (OPA-C; 2n = 70/FN = 72). An asterisk indicates a centromere; “H” indicates a large block of constitutive heterochromatin.

The karyotype of *O*. *paricola* cytotype A (OPA-A) has 2n = 72 and FN = 75, with autosomes comprising 32 acrocentric pairs (pairs 1 to 32), two meta/submetacentric pairs (pairs 33 and 34) and one heteromorphic pair (pair 35; submetacentric and acrocentric homologue). The X chromosome is a large submetacentric and the Y chromosome is a medium submetacentric (Fig 2A). The karyotype of *O*. *paricola* cytotype B (OPA-B) has 2n = 70 and FN = 75 with autosomes comprising 30 acrocentric pairs (pairs 1 to 30), three meta/submetacentric pairs (pairs 31 to 33) and one heteromorphic pair (pair 34; submetacentric and acrocentric homolog); the X chromosome is a large submetacentric, and the Y chromosome is a medium submetacentric (Fig 2B). The karyotype of *O*. *paricola* cytotype C (OPA-C) has 2n = 70 and FN = 72 with autosomes comprising 32 acrocentric pairs (pairs 1 to 32) and two meta/submetacentric pairs (pairs 33 and 34); the X chromosome is a large submetacentric, and the Y chromosome is a medium submetacentric (Fig 2C).

**Fig 3:**
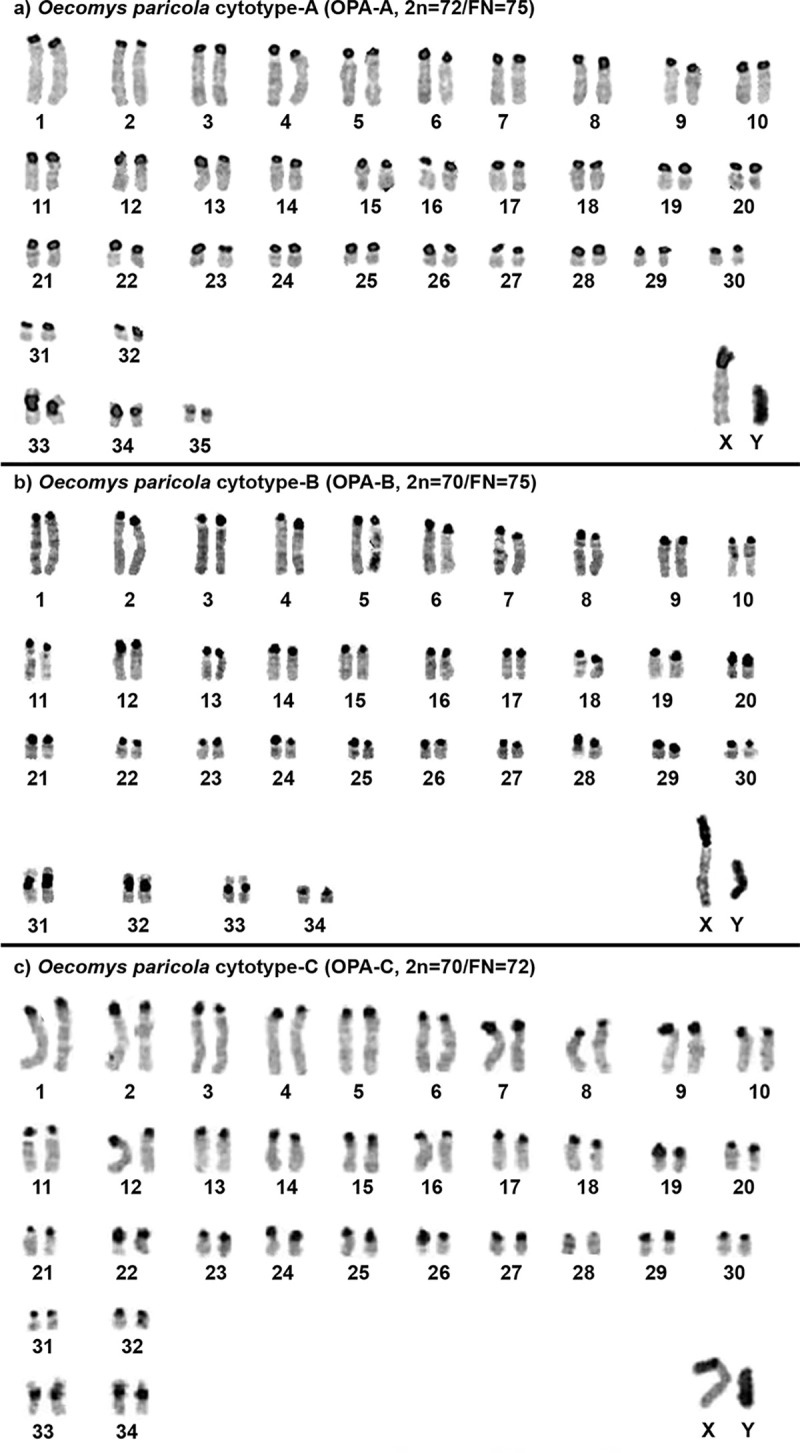
C-banded karyotypes of (a) *O*. *paricola* cytotype A (OPA-A; 2n = 72/FN = 75), (b) *O*. *paricola* cytotype B (OPA-B; 2n = 70/FN = 75) and (c) *O*. *paricola* cytotype C (OPA-C; 2n = 70/FN = 72).

The constitutive heterochromatin is distributed at the centromeric regions of almost all autosomes, in all three cytotypes; two autosomal pairs carry large heterochromatic blocks on the short arms of: OPA-A 33 and 34, OPA-B 31 and 32, OPA-C 33 and 34; the X chromosome carries a large heterochromatic block on the short arm, and the Y chromosome is almost entirely heterochromatic (Fig 3).

### Molecular cytogenetics

Chromosome painting with all 24 *Hylaeamys megacephalus* (HME) whole-chromosome probes were performed on *Oecomys paricola* cytotypes A (OPA-A; 2n = 72/FN = 75), B (OPA-B; 2n = 70/FN = 75) and C (OPA-C; 2n = 70/FN = 72), and yielded 41, 39 and 40 hybridization signals, respectively. No hybridization signals were obtained on heterochromatic regions, as well on the Y chromosome and the short arm of the X chromosome. Table 2 summarizes these results.

**Table 2:** FISH signals detected for *O*. *paricola* cytotype A (OPA-A; 2n = 72/FN = 75), *O*. *paricola* cytotype B (OPA-B; 2n = 70/FN = 75) and *O*. *paricola* cytotype C (OPA-C; 2n = 70/FN = 72), as assessed based on hybridization with HME whole-chromosome probes [22].

HME	OPA-A	OPA-B	OPA-C
**1**	2, 4, 29	2, 4, 27	2, 4, 30
**2**	9, 10	9, 10	9, 10
**3**	5, 31	5, 28	5, 31
**4**	1, 16q prox.	1, 17q prox.	1, 16q prox.
**5**	15, 22, 24	16, 21, 23	15, 22, 24
**6**	3	3	3
**7**	6	6	6
**8**	11	11	11
**(9,10)**	7, 12	7, 12	7, 12
**11**	20q dist., 28, 30, 32	13	20q dist., 27, 29
**12**	13	14	13
**(13,22)**	8q dist., 14q prox.	8q dist., 15q prox.	8q dist., 14q prox.
**14**	23, 27q dist.	22, 25q dist.	23, 28q dist.
**15**	17	26, 33	17
**(16,17)**	33, 34	31, 32	33, 34
**18**	26	24	26
**19**	16q dist., 27q prox.	17q dist., 25q prox.	16q dist., 28q prox.
**20**	8q prox., 20q prox.	8q prox.	8q prox., 20q prox.
**21**	14q dist.	15q dist.	14q dist.
**23**	19, 21	19, 20	19, 21
**24**	18	18	18
**25**	25	29, 30	25
**26**	35 (h)	34 (h)	32
**X**	Xq	Xq	Xq

Short arm (p). Long arm (q). Proximal (prox). Distal (dist). Heteromorphic pair (h).

From the 24 HME whole chromosome probes, seven (HME 6, 7, 8, 12, 18, 24 and 26) hybridized to whole chromosomes and one (HME 21) hybridized to part of one chromosome on the three OPA cytotypes; eleven probes showed multiple signals, from them nine (HME 2, 3, 4, (9,10), (13,22), 14, (16,17), 19 and 23) hybridized to two chromosomes each, and two (HME 1 and 5) showed signals in three chromosomes each on the three OPA cytotypes; the HME X chromosome hybridized to Xq due to the presence of a large heterochromatic block at Xp (Fig 2, Table 2).

The remaining four HME probes showed varied hybridization patterns among the three OPA cytotypes: HME 15 and 25 hybridized to whole chromosomes of OPA-A and OPA-C, while in OPA-B these probes hybridized on two chromosomes each; HME 20 showed multiple signals and hybridized to parts of two chromosomes each on OPA-A and OPA-C, while in OPA-B hybridized to part of one chromosome; HME 11 hybridized to a whole chromosome on OPA-B, and it is fragmented into three blocks on OPA-C and four blocks on OPA-A (Fig 2, Table 2).

**Fig 4:**
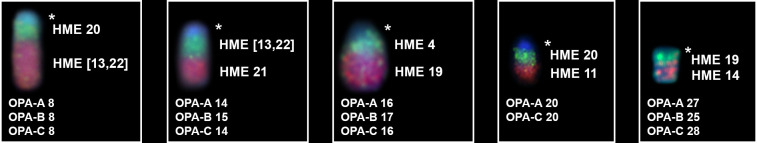
FISH results using HME whole-chromosome probes obtained from *O*. *paricola* cytotype A (OPA-A; 2n = 72/FN = 75), *O*. *paricola* cytotype B (OPA-B; 2n = 70/FN = 75) and *O*. *paricola* cytotype C (OPA-C; 2n = 70/FN = 72). Each box corresponds to an OPA chromosomal pair that hybridized to more than one HME. The identification of HME probes are shown beside the chromosomes; cytotypes and numbers from the respective chromosomal pair are shown below the chromosomes. An asterisk indicates a centromere. HME whole-chromosome probes are shown in green (FITC) and red (CY3); the counterstaining is blue (DAPI).

All three OPA cytotypes exhibited four chromosomal pairs that corresponded to more than one HME homeolog: HME 20/(13,22), (13,22)/21, 4/19 and 19/14. Another chromosomal pair that exhibited the HME 20/11 is shared only by OPA-A and OPA-C (Fig 4).

## Discussion

### Chromosomal variability in *Oecomys paricola*

Rosa et al. [26] used chromosomal banding and Ag-NOR techniques to describe two karyotypes with 2n = 68, FN = 72 and 2n = 70, FN = 76 for *O*. *paricola* samples collected at the Utinga Reserve (Table 1). Here, we used C-banding, G-banding and FISH with HME probes to reanalyze the same samples previously assigned with 2n = 68, FN = 72, and found that the correct karyotype is 2n = 72, FN = 75. We did not have access to the chromosomal preparation of samples with 2n = 70, FN = 76, and thus were unable to confirm this karyotype. Thus, we will only consider the karyotype from our study with 2n = 72, FN = 75 as representative of samples from Utinga Reserve. We designate this karyotype (Table 1, locality 2) as *O*. *paricola* cytotype A (OPA-A). We also describe two other cytotypes: *O*. *paricola* cytotype B (OPA-B; 2n = 70, FN = 75) from Santa Bárbara and Barcarena (Table 1, localities 5 and 6, respectively); and *O*. *paricola* cytotype C (OPA-C; 2n = 70, FN = 72) from Marabá (Table 1, locality 7).

The comparative analysis of chromosomal number (2n) and morphology (FN) among samples of *O*. *paricola* from distinct localities of the Amazon and Cerrado biomes (Table 1) showed that these taxa exhibit only two diploid numbers (70 and 72), with variability seen in the FN (72, 74, 75 and 76). The differences in 2n could be caused by fusion/fission events, while those in the FN could be caused either by pericentric inversions or centromeric repositioning [34]. We detected one heterozygous pericentric inversion by the presence of a heteromorphic pair with submetacentric and acrocentric homolog in OPA-A 35 and OPA-B 34; this is responsible for FN = 75, and is absent from its counterpart in the acrocentric pair 32 of OPA-C and in other *O*. *paricola* karyotypes.

Differences in the sizes of the X and Y chromosomes were observed among *O*. *paricola* samples. Suarez-Villota et al. [4] described the X and Y chromosomes of karyotypes with 2n = 70, FN = 74, 76 as “slightly heterochromatic”. However, our C-banding results showed large heterochromatic blocks at the short arms of the X chromosomes, whereas the Y chromosomes were almost entirely heterochromatic. The three OPA cytotypes also exhibited two bi-armed pairs with heterochromatic blocks (Fig 3). These variations in the sex and autosomal chromosomes were most likely caused by amplification/deletion of constitutive heterochromatin; this is a frequent event in rodents [18,35] but is unlikely to be involved in the speciation process, since heterochromatin usually does not contain functional genes and would not have a reproductive impact or generate deleterious meiotic products [36].

### Speciation hypothesis in the “*paricola* group”

**Fig 5:**
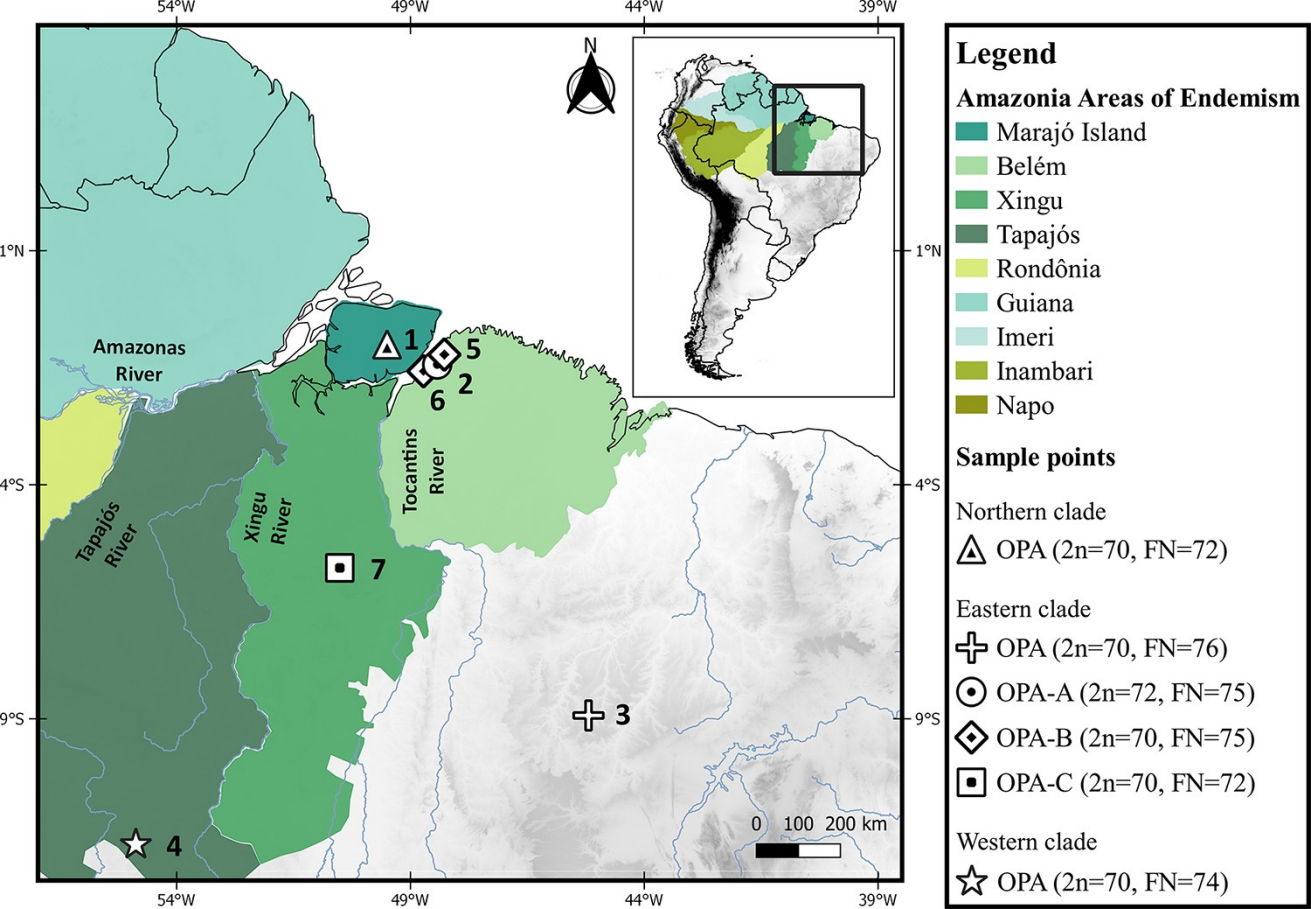
Map showing the Amazon areas of endemism recognized for terrestrial vertebrates [37], the Marajó Island, and sampling points for *Oecomys paricola* with available cytogenetic data from the literature and the present study. 2n (diploid number), FN (autosomal fundamental number) and the clades identified for *O*. *paricola* (northern, eastern and western; sensu [4]) are also shown. The localities mentioned are: Tauarí farm, Chaves, Marajó Island—PA (locality 1); Utinga Reserve, Belém—PA (locality 2); Uruçui-Una—PI (locality 3); Cláudia—MT (locality 4); Expedito Ribeiro Community, Santa Bárbara–PA (locality 5); Barcarena–PA (locality 6); Tapirapé-Aquiri National Forest, Marabá –PA (locality 7). All localities are from Brazil, and from the states of Mato Grosso (MT), Pará (PA), and Piauí (PI). The map was made using QGIS v. 3.10.7. The database was obtained from DIVA-GIS [7]. The Amazon Areas of Endemism limits were the same proposed by Silva et al. [38] (DOI: http://dx.doi.org/10.4025/bolgeogr.v34i3.30294). We used the limits provided by Silva et al. [38] and created the shapefiles on QGIS software v. 3.10.7.

Observing the distribution, karyotypic, molecular and morphological data of *O*. *paricola* from the literature and the present study can suggest insights into how the speciation process may have acted in the “*paricola* group”. The literature shows that this taxon occurs in six out of eight Amazon areas of endemism recognized for terrestrial vertebrates: the taxon is present in the Belém, Xingu, Tapajós, Rondônia, Inambari, and Napo areas of endemism, as well as in the Marajó Island [1,37]. The role of Amazonian rivers as a barrier to species distribution was proposed by Wallace [38]. Since then, many studies in terrestrial vertebrates as rodents, primates and birds have shown that the more significant rivers of the Amazon basin can act as allopatric barriers to gene flow and contribute to species diversification in Amazonia [6,20,37,39,40]. This could explain the morphological differences found between the eastern and western clades of the “*paricola* group” mentioned by Suárez-Villota et al. [4], as differences in craniodental measurements, pelage coloration and morphology of the incisive foramen and subsquamosal fenestra, since the former clade occurs in the Belém and Xingu areas of endemism, while the latter occurs in the Tapajós area of endemism [4,37] (Fig 5).

**Fig 6:**
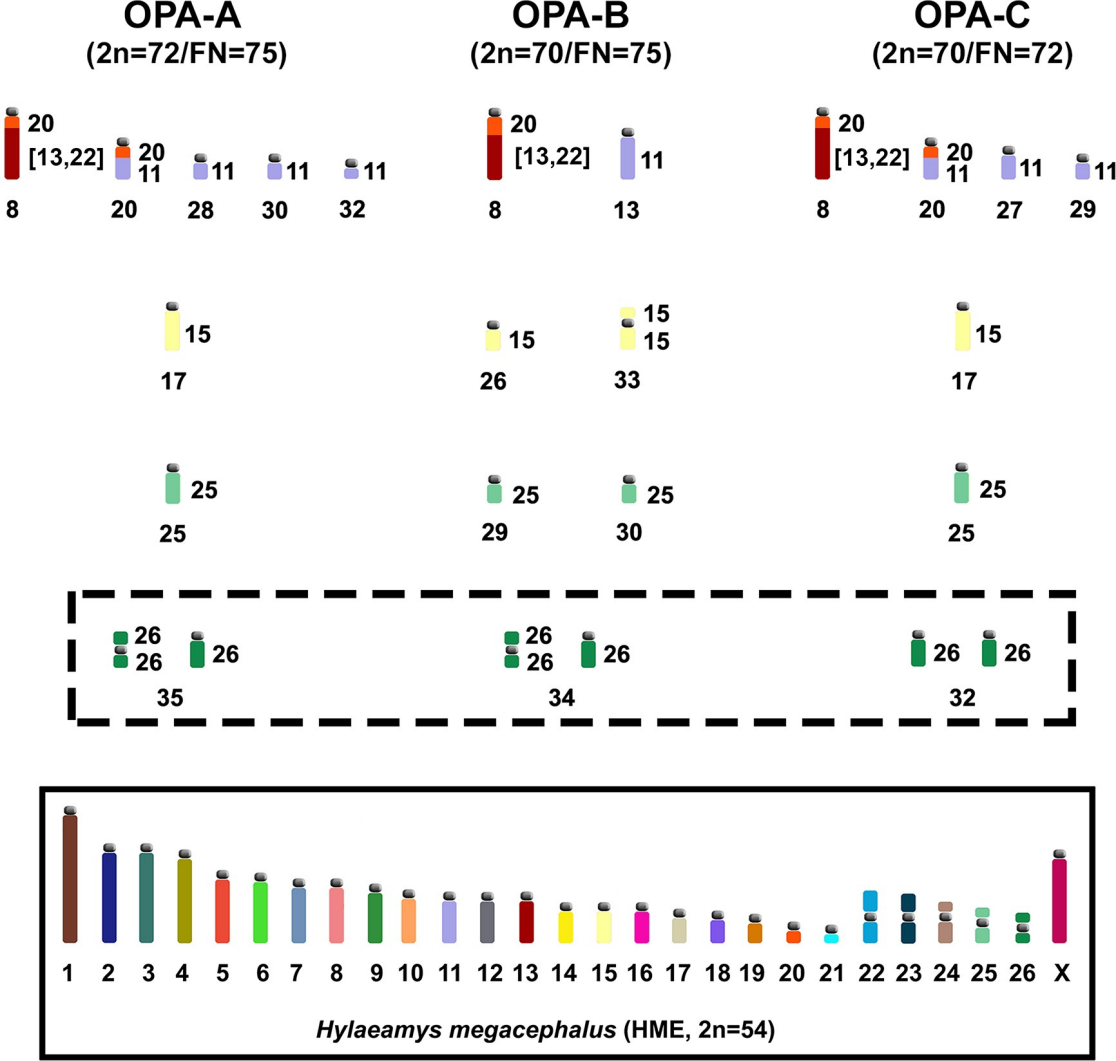
Idiograms of karyotypic differences in the haploid contents of *O*. *paricola* cytotype A (OPA-A; 2n = 72/FN = 75), *O*. *paricola* cytotype B (OPA-B; 2n = 70/FN = 75) and *O*. *paricola* cytotype C (OPA-C; 2n = 70/FN = 72), as assessed based on HME whole-chromosome probes [22]. The identification of HME probes is shown beside the idiograms; the identification of the chromosomal pairs is shown below the idiograms. Idiograms within dashed lines correspond to the diploid content. The box encompasses an idiogram of the HME karyotype elaborated by Oliveira da Silva et al. [12].

Although no significant morphological or molecular difference was found within the eastern clade [4], our comparative chromosome painting analysis showed that OPA-A (2n = 72, FN = 75) and OPA-C (2n = 70, FN = 72) diverged due to one fusion/fission event and one heterozygous pericentric inversion. This was identified based on the presence of a heteromorphic pair in OPA-A (pair 35) that is also present in OPA-B (pair 34). OPA-B (2n = 70, FN = 75) differs from OPA-A and OPA-C by five and four fusion/fission events, respectively, and one translocation (Fig 6).

Although OPA-A and OPA-C are differentiated by a few chromosomal rearrangements, they occur in distinct areas of endemism: The former is found in the Belém area of endemism and the latter in the Xingu area of endemism; they are separated by the Tocantins River, which could act as a barrier to the distribution and gene flow between these two taxa. The sample collection points of OPA-A and OPA-B were located at isolated points in the metropolitan region of Belém, where only 30% of the original forest cover remains [41] (Fig 5). The karyotypic differences discussed above between these two apparently sympatric species, the lack of a strong geographic barrier and the absence of heterozygous karyotypic forms indicate that there is no gene flow between them.

Similar results were found by Rocha et al. [5], who analyzed the *Cytb* genetic structure of *Oecomys* aff. *roberti* (= *O*. *tapajinus*) populations from the mid-Araguaia River in central Brazil, with the aim of testing how the river influenced their locomotion habits, habitat preferences and gene flow. The authors found a correlation between genetic and geographic distances, as this taxon exhibits stable and isolated populations, but did not observe any genetic difference related to the opposite riverbanks. This indicates that the isolation of *Oecomys* taxa can occur in the absence of a strong geographic barrier.

Da Silva et al. [11] discussed some biological features of rodents such as a high reproductive rate, the birth of several individuals per gestation in a short period, and a low vagility that favors endogamy that may allow the formation of an assemblage of taxonomically closely related individuals, denominated “demes” [42]. In a scenario that the same rearranged chromosomal form arises in different individuals within a population, these features could increase the probability of interbreeding between these heterozygotes. Thus, in a few generations a homozygous subpopulation for this rearranged form could arise [11].

The leading role of chromosomal rearrangements in the speciation of rodents [43] has been discussed in various studies [11,12,44,45]. Fusion/fission events and pericentric inversions are cited as the main events in the chromosomal reorganization of rodents [43], and translocations are also thought to be a strong barrier for hybridization in nature leading to diversification and speciation, as reported for rodents of genus *Ellobius* [21]. The occurrence of chromosomal rearrangements in allopatric subpopulations could act as a barrier to gene flow in a secondary contact caused by the geographic expansion of the new chromosomal forms [46]. Thus, chromosomal rearrangements could act as post-zygotic blockage of gene flow and play a leading role in the speciation process, potentially explaining the sympatry occurring between OPA-A and OPA-B, but in OPA-C the allopatric effect caused by the Tocantins River would relegate chromosomal rearrangements to a secondary role in the speciation process.

The pattern we found for OPA-A, OPA-B, and OPA-C three distinct cytotypes was not reflected in the *Cytb* cladogram presented by Suárez-Villota et al. [4], who recovered the *O*. *paricola* eastern clade as a single entity. We note that the authors of the prior study did not include any specimen from the Tapirapé-Aquiri region (Fig 5, locality 7), and thus their topology may not include OPA-C. However, the authors did include *Cytb* sequences that correspond to one of the three cytotypes recognized here from the Utinga Reserve (OPA-A, 2n = 72, FN = 75; Fig 5, locality 2) and also from Barcarena (Fig 5, locality 6), which were obtained from GenBank and originally described by Rosa et al. [26]. Although Suárez-Villota et al. [4] did not provide the specimen karyotype from Barcarena, we karyotyped samples from this locality and from Santa Bárbara (Fig 5, locality 5) that exhibit 2n = 70, FN = 75 (OPA-B). We conclude that the OPA-A and OPA-B karyotypes were represented in the *Cytb* phylogeny from Suárez-Villota et al. [4] and that the chromosomal divergences described herein were not reflected in the *Cytb* sequence data.

In summary, our results indicate that OPA-A, OPA-B and OPA-C are three distinct species that belong to the *O*. *paricola* eastern clade (sensu [4]), with sympatry occurring between OPA-A and OPA-B. In this sense, the “*paricola* group” is more diverse than was reported previously, and a review of taxonomy of this group is needed to fully address the geographical limits and taxonomic delimitations. Lastly, detailed phylogeographic studies are necessary to improve our understanding of the speciation process in the genus *Oecomys*.

### Chromosomal signatures for *Oecomys*

Previously published studies using HME whole-chromosome probes allowed the proposition of chromosomal signatures for the Sigmodontinae subfamily (HME 7/(9,10), 8, 1/12, 6/21, 11/(16,17), 5/(16,17), 20/(13,22), 15, 19/14/19, 24, and 26) and the Oryzomyini tribe (HME 8a, 8b, 18, and 25) [2,12,19,20,22–24]. We herein performed a comparative chromosome painting analysis with those data (S1 Table) and our findings are consistent with the above proposals.

Regarding genus *Oecomys*, only *O*. *catherinae* from Pará (OCA-PA; 2n = 62, FN = 62) and *O*. *catherinae* from Rio de Janeiro (OCA-RJ; 2n = 60, FN = 62) had previously been analyzed by chromosome painting with HME probes [2]. Our comparative analysis among OPA-A, OPA-B, OPA-C, OCA-PA and OCA-RJ karyotypes revealed the following specific signatures for this group: the syntenic block HME (13,22)/21 and the fragmentation of HME 1 into three blocks are exclusive traits for the *Oecomys* genus; HME (9,10)/14/5, 23/19/11 and 26/11 are exclusive traits for OCA-PA and OCA-RJ; and HME 4/19 and the fragmentation of HME 3 into two blocks are exclusive traits of OPA-A, OPA-B and OPA-C.

Although the syntenic block HME 20/(13,22) is considered to be an ancestral trait of the Sigmodontinae, in both OPA-A and OPA-C it is present as a derived form; this is due to a translocation that generates the syntenic block HME 20/(13,22) and 20/11, which are exclusive traits for OPA-A and OPA-C.

In the future, the use of comparative chromosome painting in other *Oecomys* species could help improve the taxonomic delineation, particularly in those taxa that are proposed to constitute species complexes (e.g., *O*. *bicolor*, *O*. *catherinae*, *O*. *cleberi*, *O*. *mamorae*, *O*. *paricola* and *O*. *roberti*) and in which morphological and/or molecular methods could not fully establish species boundaries.

## Conclusions

Our comparative chromosome painting analysis show that OPA-A, OPA-B and OPA-C differ by fusion/fission events, translocations and pericentric inversions (or centromeric repositionings), and allow the detection of chromosomal signatures that can be used as phylogenetic markers for genus *Oecomys* and species *O*. *paricola* and *O*. *catherinae*. Our results also indicate that OPA-A, OPA-B and OPA-C are three distinct species that belong to the eastern clade, with sympatry occurring between OPA-A and OPA-B. Moreover, we suggest that chromosomal rearrangements have played a leading role in the speciation process of OPA-A and OPA-B, but in OPA-C the allopatric effect caused by the Tocantins River would relegate chromosomal rearrangements to a secondary role, and that the *Oecomys paricola* complex is more diverse than was previously alleged.

## Supporting information

S1 Table FISH signals detected for Sigmodontinae species, as assessed based on hybridization with *Hylaeamys megacephalus* (HME) whole-chromosome probes [22]. (DOCX)
