# Supplementary material for: Karyotypic divergence reveals that diversity in the Oecomys paricola complex (Rodentia, Sigmodontinae) from eastern Amazonia is higher than previously thought
Source: PLoS One. 2020 Oct 29;15(10):e0241495. doi: 10.1371/journal.pone.0241495 (PMC7595413; doi:10.1371/journal.pone.0241495)
Supplement: S1 Table — (DOCX) [file pone.0241495.s001.docx]

**Supplementary Table 1. FISH signals detected for Sigmodontinae species, as assessed based on hybridization with *Hylaeamys* *megacephalus* (HME) whole-chromosome probes [22].**

| HME | CLA | OCA-PA | OCA-RJ | OPA-A | OPA-B | OPA-C | NSP-A | NSP-B | NSP-C | NSP-D | NPA | NSP-E | NAM | TNI | AMO | ASP | NLA | OAM | BBR |
| --- | --- | --- | --- | --- | --- | --- | --- | --- | --- | --- | --- | --- | --- | --- | --- | --- | --- | --- | --- |
| 1 | 2q, 20 | 13, 16, 29 | 13, 17, 28 | 2, 4, 29 | 2, 4, 27 | 2, 4, 30 | 6, 8 | 2p, 4q | 6, 8 | 6, 8 | 6, 8 | 6, 8 | 6, 8 | 4, 8 | 1q dist., 4q | 1q int., 2q dist. | 5q dist., 7 | 12, 13 | 2p, 4q |
| 2 | 10, 18, 19 | 4 | 1p, 4 | 9, 10 | 9, 10 | 9, 10 | 2 | 3q | 2 | 2 | 2 | 2 | 2 | 7, 12 | 1q int., 7q | 1p dist., 2q int. | 9q int., 13 | 8 | 5q |
| 3 | 1q int., 3p | 1 | 1q | 5, 31 | 5, 28 | 5, 31 | 3 | 2p | 3 | 3 | 3 | 3 | 3 | 1 int. and dist. | 2q | 2p dist. | 3q dist. | 1q dist. | 1q dist. |
| 4 | 5, 13 | 2 | 2 | 1, 16q prox. | 1, 17q prox. | 1, 16q prox. | 1q dist. | 1q dist. | 1q dist. | 1q dist. | 1q dist. | 1q dist. | 1q dist. | 13, 15 | 1p prox., 5p dist. | 2q prox. and int., 3q int. | 1q prox., 10q prox., 11q dist. | 9, 24 | 3q |
| 5 | 1p dist., 1q prox., 8 | 3q dist., 15, 27 | 3q dist., 15, 27 | 15, 22, 24 | 16, 21, 23 | 15, 22, 24 | 19, 22, 24, 26p | 3p dist., 21, 22, 24 | 15q dist., 19, 24 | 15q dist., 19, 23 | 19, 26 | 9, 17 | 9, 28, 31 | 2 dist., 5 prox., 6 prox. | 3q int., 6p int., 10 | 1p int. and prox., 3q int. (ts) | 6q dist., 12q int., 14q dist. | 2p prox., 3p, 23, 25 | 7q, 9q dist. |
| 6 | 4q dist. | 9, 25, 26 | 9, 16 | 3 | 3 | 3 | 5q prox., 18 | 5q prox., 8 | 5q prox., 18 | 5q prox., 18 | 5q prox., 18 | 5q prox., 19 | 5q prox., 18 | 3 prox. and int. | 2p | 2p int. | 2q int. | 7q prox. | 2q prox. |
| 7 | 3q int. | 7 | 7 | 6 | 6 | 6 | 7, 9q int. | 3p int., 5p | 7, 9q int. | 7, 9q int. | 7, 9q int. | 7, 10q int. | 7, 10q int. | 18 | 5q prox., 8q | 1p int., 3q int. | 1q int., 4q prox. | 11q prox., 15 | 1p prox., 9p |
| 8 | 4q prox., 7 | 5 | 5 | 11 | 11 | 11 | 12, 13 | 6, 15 | 12, 13 | 12, 13 | 12, 13 | 12, 16 | 12, 16 | 6 dist. | 3p | 3q prox. | 1q int. | 10 | 4p prox., 6q |
| (9,10) | 2p dist., 3q dist. | 3q prox., 12 | 3q prox., 12 | 7, 12 | 7, 12 | 7, 12 | 9q (ts), 10 | 1p, 3p int. (ts) | 9q (ts), 10 | 9q (ts), 10 | 9q (ts), 10 | 10q (ts), 14 | 10q (ts), 14 | 2 prox., 5 dist. | 5q, 9p | 1q int., 3q int. | 1q dist., 6q prox. | 11q dist., 17 | 1p dist., 8p |
| 11 | 11q prox., 6 | 6q dist., 8 dist. | 6q dist.; 8q dist. | 20q dist., 28, 30, 32 | 13 | 20q dist., 27, 29 | 20, 23p | 11, 26 | 20, 22 | 20, 22q | 14, 25p | 24, 25 | 22, 23 | 9 dist., 10 prox. | 1p int., 6q dist. | 2q int., 3q dist. | 10q int., 12q dist. | 2q dist., 16q prox. | 3p dist. |
| 12 | 2p prox. | 14 | 14 | 13 | 14 | 13 | 4q prox. | 4q prox. | 4q prox. | 4q prox. | 4q prox. | 4q prox. | 4q prox. | 16 | 1q int. | 2q int. | 5q prox. | 18 | 8q |
| (13,22) | 1q (ts), 9 | 10q dist., 18q prox., 22 | 10q dist., 19q prox., 22 | 8q dist., 14q prox. | 8q dist., 15q prox. | 8q dist., 14q prox. | 1q int., 26q, 27p | 1q int., 19, 23 | 1q int., 21, 28p | 1q int., 21, 28p | 1q int., 20, 21 | 1q int., 21, 23 | 1q int., 19, 21 | 9 prox., 11 int. and dist., 21 | 3q prox., 4p dist., 6q prox. | 1q int., 3q int. (ts) | 4q dist., 12q prox., 14q prox. | 2p dist., 3q, 14q dist. | 5p dist., 9q prox., 12 |
| 14 | 1p int., 21 | 3q int., 30 | 3q int., 29 | 23, 27q dist. | 22, 25q dist. | 23, 28q dist. | 23q, 25p prox. | 20, 25 | 25q, 26p prox. | 24q, 25p prox. | 16, 24p prox. | 26, 28 | 24, 26 | 17 prox., 24 | 6p prox., 8p int. | 1p int., 3q int. | 2q int., 15q int. | 21q int., 26 | 7p proximal, 13 |
| 15 | 12 | 11 | 11 | 17 | 26, 33 | 17 | 15 | 9 | 17 | 17 | 15 | 15 | 15 | 19 | 9q | 1q dist. | 8q dist. | 19 | 6p |
| (16,17) | 1q prox., 11q dist. | 19, 20 | 20, 24 | 33, 34 | 31, 32 | 33, 34 | 4q dist., 16 | 4q dist., 10 | 4q dist., 16 | 4q dist., 16 | 4q dist., 25q | 4q dist., 22 | 4q dist., 20 | 10 dist., 22 | 1p dist., 3q dist. | 2p prox., 2q int., 3q int. | 2q prox., 10q dist. | 4, 16q dist. | 4p dist., 10p |
| 18 | 16 | 21 | 21 | 26 | 24 | 26 | 17 | 7 | 15q prox., | 15q prox. | 17 | 27 | 25 | 1 prox., 23 | 1q prox., 5p prox. | 2p int., 2q int., 3q int. | 3q prox., 9q prox. | 1p prox., 5 | 3p prox.,  10q |
| 19 | 1p int., 3q prox. | 6q int., 28 | 6q int., 26 | 16q dist., 27q prox. | 17q dist., 25q prox. | 16q dist., 28q prox. | 14, 25p dist. | 18 | 14, 26p dist. | 14, 25p dist. | 24p dist., 24q | 18 | 17 | 17 dist. | 8p dist., 8q prox. | 1p int. (ts) | 15q prox. and dist. | 21q (prox. and dist.) | 7p dist. |
| 20 | 1q dist. | 10q prox. | 10q prox. | 8q prox., 20q prox. | 8q prox. | 8q prox., 20q prox. | 1q prox. | 1q prox. | 1q prox. | 1q prox. | 1q prox. | 1q prox. | 1q prox. | 11 prox. | 4q prox. | 1q prox. and int. | 4q int. | 14q prox. | 5p prox. |
| 21 | 4p, 4q int. | 18q dist. | 19q dist. | 14q dist. | 15q dist. | 14q dist. | 5q dist. | 5q dist. | 5q dist. | 5q dist. | 5q dist. | 5q dist. | 5q dist. | 3 dist. | 2p dist. | 2p int. | 2q dist. | 7q dist. | 2q dist. |
| 23 | 15 | 6q prox., 23 | 6q prox., 23 | 19, 21 | 19, 20 | 19, 21 | 21, 25q | 14, 16 | 23, 26q | 25q, 26q | 23 | 13, 20 | 13, 29 | 20 | 7p | 1p int. | 8q prox. | 20 | 3p int. |
| 24 | 14 | 17 | 18 | 18 | 18 | 18 | 11 | 12 | 11 | 11 | 11 | 11 | 11 | 14 | 6p dist. | 3q int. (ts) | 9q dist. | 22 | 11q |
| 25 | 17 | 24 | 25 | 25 | 29, 30 | 25 | 28 | 13 | 27 | 27 | 27 | 29 | 27 | 1 prox. | 2p prox. | 2p int., 3q int. | 3q int., 11q prox. | 1p dist., 1q int. | 1q prox. |
| 26 | 22 | 8q prox. | 8q prox. | 35 (h) | 34 (h) | 32 | 27q | 17 | 28q | 28q | 22 | 30 | 30 | 25 | 11 | 4 | 16 | 6 | 11p |
| X | X | Xq | Xq | Xq | Xq | Xq | Xq | X | Xq | Xq | X | Xq | Xq | X | X (Xq) | X | X | Xq | X |

Short arm (p). Long arm (q). Proximal (prox). Interstitial (int). Distal (dist). Two segments (ts). Heteromorphic pair (h). *Hylaeamys megacephalus* (HME), *Cerradomys langguthi* (CLA) [22], *O. catherinae-*Pará (OCA-PA), *O. catherinae-*Rio de Janeiro (OCA-RJ) [2], *Oecomys paricola* cytotype A (OPA-A), *Oecomys paricola* cytotype B (OPA-B), *Oecomys paricola* cytotype C (OPA-C) (present study), *Neacomys* sp. A (NSP-A), *Neacomys* sp. B (NSP-B) [20], *Neacomys* sp. C (NSP-C), *Neacomys* sp. D (NSP-D), *N. paracou* (NPA), *Neacomys* sp. E (NSP-E), *N. amoenus* (NAM) [12], *Thaptomys nigrita* (TNI), *Akodon montensis* (AMO) [23], *Akodon* sp. (ASP), *Necromys lasiurus* (NLA) [24], *Oxymycterus* *amazonicus* (OAM), *Blarinomys* *breviceps* (BBR) [19].
